# Association of *PTPN22*-C1858T Polymorphism With Susceptibility to *Mycobacterium tuberculosis* and *Mycobacterium leprae* Infection: A Meta-Analysis

**DOI:** 10.3389/fimmu.2021.592841

**Published:** 2021-02-25

**Authors:** Shuping Li, Xiaohua Wang, Yuming Zhao, Juan Yang, Tianjiao Cui, Zhizhuang Joe Zhao, Yun Chen, Zhihua Zheng

**Affiliations:** ^1^ Department of Nephrology, The Seventh Affiliated Hospital, Sun Yat-Sen University, Shenzhen, China; ^2^ Center of Nephrology and Urology, The Seventh Affiliated Hospital, Sun Yat-Sen University, Shenzhen, China; ^3^ Scientific Research Center, The Seventh Affiliated Hospital, Sun Yat-Sen University, Shenzhen, China

**Keywords:** *PTPN22*-C1858T, single-nucleotide polymorphism, tuberculosis, leprosy, *Mycobacterium tuberculosis*, *Mycobacterium leprae*

## Abstract

It was previously published that single-nucleotide polymorphism rs2476601 (*PTPN22* [protein tyrosine phosphatase non-receptor type 22]-C1858T) might be related to increased sensibility to *Mycobacterium tuberculosis* and *M. leprae* infection. However, the results were inconclusive despite a high degree of similarity between both parameters. Herein, we carried out this meta-analysis to systematically summarize and articulate the correlation between *PTPN22*-C1858T polymorphism and mycobacterial infection. The susceptibility of *PTPN22*-C1858T carriers with autoimmune conditions receiving immunosuppressive therapy to *M. tuberculosis* and *M. leprae* infection was determined. A systematic retrieval of studies on relevance of *PTPN22*-C1858T polymorphism to susceptibility of *M. tuberculosis* or *M. leprae* infection was performed in Chinese National Knowledge Infrastructure, PubMed and Embase databases. We regarded Odds ratios (ORs) and 95% confidence intervals (CIs) as the determined effect size. Finally, four and two case-control studies on tuberculosis and leprosy, respectively, were included. In all genetic models, without indicated association between *PTPN22*-C1858T polymorphism and tuberculosis’s susceptibility. [C versus T: OR = 0.22 (95% CI: 0.09–0.50, P_H_ = 0.887); CT versus CC: OR = 0.21 (95% CI: 0.09–0.49, P_H_ = 0.889); TT+CT versus CC: OR = 0.21 (95% CI: 0.09–0.49, P_H_ = 0.889)]. A significantly increased risk of leprosy was perceived in patients with the *PTPN22*-C1858T polymorphism [C versus T: OR = 2.82 (95% CI: 1.02–7.81, P_H_ = 0.108)]. While the *PTPN22*-C1858T polymorphism is irrelevant to higher susceptibility to the infection of *M. tuberculosis* in Caucasians and Asians, it is relevant to increased susceptibility to the infection of *M. leprae*. However, the results of *M. leprae* are supposed to interpreted with prudence owing to the limited quantity of studies and heterogeneity. Further well-designed studies with sufficient populations are required to verify our conclusions.

## Introduction

Worldwide, tuberculosis is one of the principal lethal causes, ranking above human immunodeficiency virus infection/acquired immunodeficiency syndrome. Despite being a preventable and curable disease, the annual number of people died from tuberculosis is 1.5 million approximately. (1). Tuberculosis accounts for a great share of global burden of disease, particularly among vulnerable populations, and exerts significant impacts on 10 million people annually (2). By recent estimates, over 200,000 new leprosy cases are diagnosed annually. At the end of 2018, the prevalence rate of leprosy is 0.2/10,000 and a total of 184,212 leprosy cases has been registered (3). Leprosy can be cured using effective and affordable multidrug therapy; however, it is a major problem in resource-poor tropical and warm temperate countries and keeps endemic in several low- and middle-income countries globally (4, 5). Available evidence indicates that inferior living conditions are possible to raise the risk of leprosy (6). Moreover, the correlation between tuberculosis or leprosy and sociodemographic risk markers such as crowded living conditions, poor sanitation, and poverty has been validated across diverse geographical settings, both in ecological- and individual-level studies (4, 7).

Tuberculosis and leprosy are caused by mycobacteria (8). In humans, tuberculosis is predominantly caused by *Mycobacterium tuberculosis* and occasionally by other components of *M. tuberculosis* complex, and *Mycobacterium leprae* result in leprosy. *M. tuberculosis* and *M. leprae* are obligate pathogens with a similar morphology and high cell wall lipid content, which is responsible for their increased chromaticity, resistance, and pathogenicity. Moreover, these mycobacteria can cause chronic granuloma in most infected individuals.

Locating on chromosome 1p13, the protein tyrosine phosphatase non-receptor type 22 (*PTPN22*) gene encodes the protein lymphoid tyrosine phosphatase (LYP), which regulates the activation of protein kinases to modulate intracellular tyrosine phosphorylation incidence and induce various biological effects. *PTPN22*-C1858T is the most talked about single-nucleotide polymorphism (SNP) in the field of autoimmunity (9); this SNP causes R620W substitution in *PTPN22* gene’s C-terminal region. *PTPN22*-C1858T was identified as a missense SNP in *PTPN22* gene’s exon 14 by using the candidate gene approach in 2004. Moreover, genome-wide association studies showed a correlation between *PTPN22*-C1858T polymorphism and increased risk of autoimmune diseases (10–15) as well as bacterial infections (16). In addition to disease-causing microorganisms in the common disease spectrum, the susceptibility of individuals carrying *PTPN22*-C1858T SNP to tuberculosis and leprosy has been proposed in preceding studies (17–19).

Herein, we performed this meta-analysis to systematically summarize and articulate the correlation between *PTPN22*-C1858T polymorphism and the risk of mycobacterial infection.

## Methods

### Retrieval of Studies

Two investigators searched literatures from Chinese National Knowledge Infrastructure (CNKI), PubMed and Embase databases independently and systematically, which were updated on April 15, 2020. Retrieved items including “tuberculosis or (pulmonary tuberculosis) or (mycobacterium tuberculosis) or (tubercle bacillus) or (tubercle bacillus) or mtb)”, “leprosy or (leprosy bacillus) or (mycobacterium leprae) or (mycogerms leprae) or (wholemycobacterium leprae)”, “PTPN22 OR LYP”, “polymorphism OR poly-morphism” and susceptibility” were searched in the National Institutes of Health (PubMed) and European (Embase) databases without limitation. The key words “PTPN22基因多态性” and “结核” were searched in the CNKI database. The references in retrieved studies and related reviews were also under reviewing by two investigators without communications.

### Inclusion Criteria and Excluding Criteria

All studies pertaining to *PTPN22* polymorphisms and infection susceptibility were electronically retrieved. Thereafter, case-control studies were identified from their titles and abstracts. The full article of manuscripts fulfilled the inclusion criteria was perused. Inclusion criteria were as follows: (a) studies associated between *PTPN22* polymorphisms and infection susceptibility; (b) the category of the study is case-control study among human; (c) original research with detailed explanation of the sample size; (d) with odds ratios (ORs) and its corresponding 95% confidence intervals (CIs); (e) with clear determination of genotype frequency; (f) genotype distribution in controls complying with Hardy-Weinberg equilibrium (HWE) law. Excluding criteria were as follows: (a) non-conformance to the inclusion criteria; (b) article type: abstract, review, comment, and letter; (c) studies with insufficient or no explanation of the sample size; (d) studies without genotype data; (e) repeated publications; (f) family-based studies. If several studies were with same population, the larger one will be included. According to the inclusion criteria and excluding criteria, two investigators screened out eligible literatures independently through titles, abstracts, and full-texts. We settled all dissents through discussion. The third researcher was consulted in the absence of an agreement. Finally, two studies were discussed and excluded after our discussions.

### Data Withdrawal

Two investigators withdrawn the data from all included studies independently. The extracted data included the name of first author, year of publication, subject resource, ethnicity, infectious type, diagnostic criteria for infection, genotyping method, number of participants in the control and case groups, cases’ and controls’ characteristics, all subjects’ *PTPN22*-C1858T genotype frequency and HWE. Therein, populations from all included studies were classified into two ethnic: Caucasians and Asians. Two investigators verified the withdrawn data and achieved agreements on the conclusive data. In the case of a disagreement, the original data was re-verified and re-discussed to reach consensus. If disagreements are still subsistent, we consulted for final judgement from a third investigator. In the end, a study was discussed through third investigator.

### Literature Assessment

The Newcastle-Ottawa Scale (NOS) was applied to assess the quality of six eligible studies in meta-analysis by two independent investigators (20) (Methods were shown in **Supplement 2**). All study were assessed by using a “star system” on the basis of three categories: selection of case and control, subjects’ comparability, and exposure’s ascertainment. The maximum score is 9 on the NOS scale. If a study with a score ≥ 7, it is considered as a high-quality study. The details of assessment has been shown in **Table S3**.

### Statistical Methods

The PRISMA checklist was followed throughout the course of this meta-analysis strictly. (21). The HWE was used to assess genotyping errors in control groups by Chi-squared test in all studies (*P* < 0.05 was significant). ORs and its corresponding 95% CI is was applied to determine the extent of correlation between *PTPN22*-C1858T polymorphism and susceptibility to mycobacterial infection. We calculated the pooled ORs in allelic comparison (*PTPN22*: C versus T), the heterozygote model (*PTPN22*: CT versus CC), and the dominant model (*PTPN22*: CT+TT versus CC). *P* < 0.05 was significant according to Z-test. Heterogeneity was assessed by Q test (*P* < 0.1 was significant) and I-squared statistic (I^2^: 0%–25%, without heterogeneity; I^2^: 25%–50%, moderate heterogeneity; I^2^: 50%–75%, large heterogeneity; I^2^: 75%–100% extreme heterogeneity; I^2^ > 50% represented significant inconsistency in our meta-analysis) (22). We applied a fixed-effect or a random-effects model to pool effect sizes depending on data heterogeneity (23). Heterogeneity was analyzed by using meta-regression model (*P* < 0.1 was considered significant) with pathogen (*M. tuberculosis* and *M. leprae*) and ethnicity and subgroup analysis stratified by pathogen and ethnicity. No deviation from HWE was shown among controls in all included studies, which are used for further meta-analysis. Additionally, we assessed publication bias by Begg’s funnel plots (24). An asymmetric plot with *P* < 0.05 was identified a significant publication bias. Besides, we also performed sensitivity analysis to estimate the pooled ORs of *PTPN22*-C1858T polymorphism in each study. The results of the meta-analysis were recalculated after excluding each studies. All statistical analyses were performed by Stata 14.0 software (StataCorp, College Station, TX, USA). Except for certain conditions defined a specific P value, a two-tailed P value < 0.05 was regarded as significant.

## Result

### Study Retrieval

**Figure 1:**
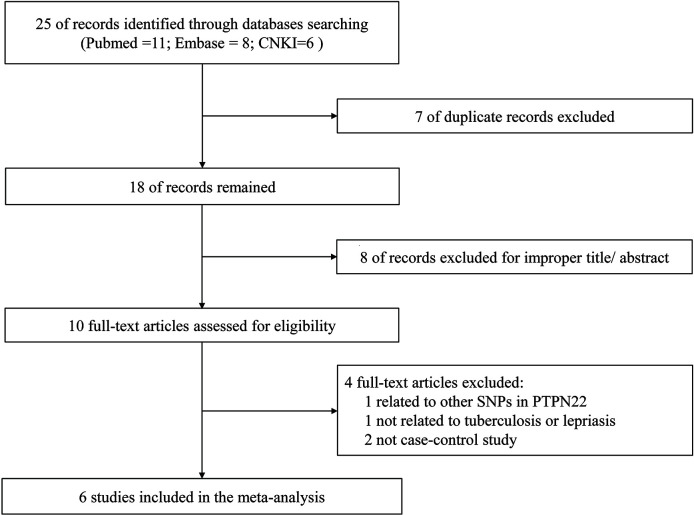
Flowchart of the studies retrieval.

The study selection process during meta-analysis of association of rs2476601(PTPN22-C1858T) polymorphism with M. tuberculosis and M. leprae infection is shown in **Figure 1**. Following the initial retrieval of 25 publications through a database search (eleven from PubMed, eight from Embase, and six from CNKI), 18 records were selected after the removal of seven duplicates. Moreover, after careful review of the title and abstract, eight publications were rejected because of their irrelevance to this meta-analysis. The remaining ten publications were full-article reviewed; of these, four were excluded. One of them was related to other SNPs in *PTPN22*, one was not related to tuberculosis or lepriasis, and two were not case-control studies. Finally, six case-control studies (17–19, 25–27) consisting of 2,160 participants (cases = 901; controls = 1,259) were included in meta-analysis. General characteristics of the six studies are in **Table 1** while the genotype distribution of subjects is shown in **Table 2**.

**Table 1:** General characteristics of studies in meta-analysis.

Study ID	Year	Country	Ethnicity	Case			Control			P value for HWE*	Quality*	Pathogen	Method of SNP	Diagnostic method
				CC	CT	TT	CC	CT	TT					
Gomez, L. M. (18)	2005	Spain	Caucasian	110	3	0	147	14	0	0.5641	8	*Mycobacterium tuberculosis*	TaqMan	Detection of acid-fast bacilli in sputum or isolation of *M. tuberculosis* in culture
Lamsyah, H. (19)	2009	Morocco	Caucasian	122	1	0	145	10	0	0.6781	8	*Mycobacterium tuberculosis*	TaqMan	Microbiological diagnosis, medical history, physical examination and chest X-ray
Shi, X. (27)	2014	China	Asian	233	2	0	240	11	0	0.7226	7	*Mycobacterium tuberculosis*	PCR	According to the guidance of China in 2002.*
Narasimha, V. R. (26)	2016	India	Asian	124	0	0	129	1	0	0.9649	6	*Mycobacterium tuberculosis*	PCR	Acid-fast bacilli in sputum samples, chest X-ray, Mantoux and fine-needle aspiration cytology tests.
Rani, R. (17)	2009	India	Asian	129	24	0	351	14	0	0.7087	7	*Mycobacterium leprae*	PCR	On the basic of immunological, histopathological, and bacteriological status.
Aliparasti, M. R. (25)	2013	Iran	Asian	146	7	0	191	6	0	0.8282	7	*Mycobacterium leprae*	PCR	According to criteria of World Health Organization.

*HWE, Hardy-Weinberg equilibrium. *Quality: According to Newcastle-Ottawa Scale (NOS); *Guidance: Guidelines for the Implementation of the Tuberculosis Prevention and Control Program in China (published by former Department of Disease Control, Ministry of Health.

**Table 2:** Summary of the pooled ORs in multiple genotypes models.

Variables	N	Allele model		Heterozygous model		Dominant model	
		C/T		CT/CC		TT+CT/CC	
		OR(95% CI)	P_H_	OR(95% CI)	P_H_	OR(95% CI)	P_H_
Overall	6	0.59(0.15, 2.29)	0.000	0.58(0.14, 2.36)	0.000	0.58(0.14, 2.36)	0.000
Pathogen							
*Mycobacterium tuberculosis*	4	0.22(0.09, 0.50)	0.887	0.21(0.09, 0.49)	0.889	0.21(0.09, 0.49)	0.889
Caucasian	2	0.22(0.08, 0.64)	0.470	0.21(0.07, 0.62)	0.474	0.21(0.07, 0.62)	0.474
Asian	2	0.21(0.05, 0.82)	0.739	0.21(0.05, 0.81)	0.733	0.21(0.05, 0.81)	0.733
* Mycobacterium leprae*	2	2.82(1.02, 7.81)	0.108	2.91(0.99, 8.60)	0.094	2.91(0.99, 8.60)	0.094
Ethnicity							
Caucasian	4	1.02(0.22, 4.70)	0.001	1.03(0.21, 4.97)	0.001	1.03(0.21, 4.97)	0.001
Asian	2	0.22(0.08, 0.64)	0.470	0.21(0.07, 0.62)	0.474	0.21(0.07, 0.62)	0.474

N, number of included studies; OR, odds ratio; CI, confidence interval.

### The Relevance Between *PTPN22*-C1858T Polymorphism and the Susceptibility to Mycobacterial Infection

**Figure 2:**
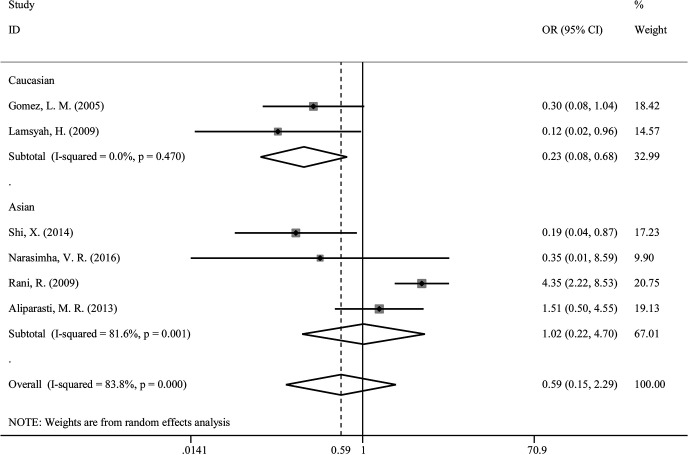
Forest plot for meta-analysis of association of rs2476601 (*PTPN22*-C1858T) polymorphism with increased susceptibility to *Mycobacterium* tuberculosis or M. leprae infection in Caucasians and Asians (C versus T). The size of blocks or diamonds is on behalf of the weight and the length of the straight line is on behalf of 95% confidence interval’s width.

We explored that *PTPN22*-C1858T polymorphism associates susceptibility to mycobacterial infection. After Q-test and I-squared statistics in various genetic models, it indicated significant heterogeneity. Therefore, we used the random-effects model in meta-analysis. No genetic model showed correlation between *PTPN22*-C1858T polymorphism and increased susceptibility to mycobacterial infection [C versus G: OR = 0.59 (95% CI: 0.15–2.29, P_H_ = 0.000) (**Figure 2**); CT versus CC: OR = 0.58 (95% CI: 0.14–2.36, P_H_ = 0.000); CT+TT versus CC: OR = 0.58, (95% CI: 0.14–2.36, P_H_ = 0.000)] (**Table 2**). Then, we further performed meta-analysis in the subgroups with different ethnicities. In Asians, no genetic model revealed correlation between *PTPN22*-C1858T polymorphism and increased susceptibility to mycobacterial infection [C versus G: OR = 0.22 (95% CI: 0.08–0.64, P_H_ = 0.470); CT versus CC: OR = 1.03 (95% CI: 0.21–4.97, P_H_ = 0.001); CT+TT versus CC: OR = 1.03 (95% CI: 0.21–4.97, P_H_ = 0.001)] (**Table 2**). In Caucasians, the result indicated that *PTPN22*-C1858T polymorphism correlates the susceptibility to mycobacterial infection [C versus G: OR = 1.02 (95% CI: 0.22–4.70, P_H_ = 0.001); CT versus CC: OR = 1.03 (95% CI: 0.21–4.97, P_H_ = 0.001); CT+TT versus CC: OR = 1.03 (95% CI: 0.21–4.97, P_H_ = 0.001)] (**Table 2**).

### *PTPN22*-C1858T Polymorphism Uncorrelated With Susceptibility to *M. tuberculosis* Infection

**Figure 3:**
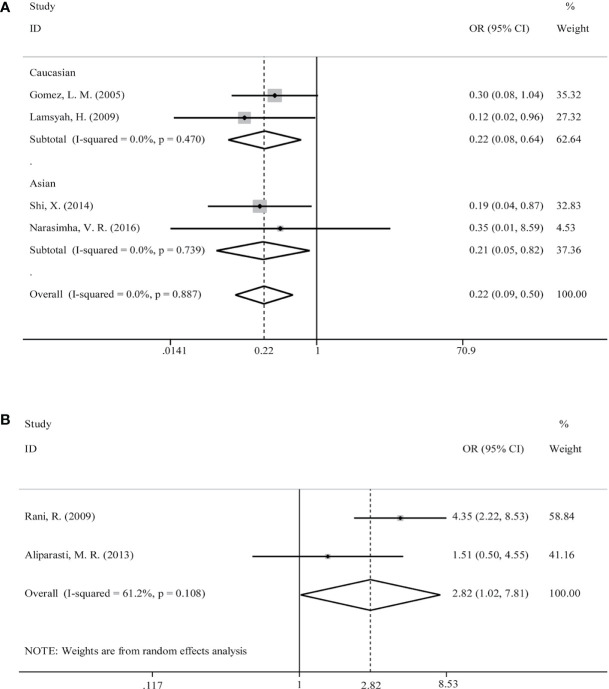
Forest plot for meta-analysis of association of rs2476601 (*PTPN22*-C1858T) polymorphism with increased susceptibility to *Mycobacterium* tuberculosis **(A)** infection in Caucasians and Asians and M. leprae **(B)** (C versus T). The size of blocks or diamonds is on behalf of the weight and the length of the straight line is on behalf of 95% confidence interval’s width.

Initially, we analyzed the correlation between *PTPN22*-C1858T polymorphism and *M. tuberculosis* infection in our included studies of *M. tuberculosis* infection. There was no genetic model supported significant correlation between *PTPN22*-C1858T polymorphism and increased possibility to M. tuberculosis infection [C versus T: OR = 0.22 (95% CI: 0.09–0.50, P_H_ = 0.887) (**Figure 3A**); CT versus CC: OR = 0.21 (95% CI: 0.09–0.49, P_H_ = 0.889); TT+CT versus CC: OR = 0.21 (95% CI: 0.09–0.49, P_H_ = 0.889)] (**Table 2**).

Then, a further meta-analysis was performed in *M. tuberculosis* infection on the four included studies according to ethnicity (18, 19, 26, 27). All three genetic models showed significant homogeneity among Caucasians; therefore, a fixed-effects model was applied. However, all of involved genetic models didn’t illustrate an association with significance between *PTPN22*-C1858T polymorphism and increased susceptibility to mycobacterial infection in Caucasians [C versus T: OR = 0.22 (95% CI: 0.08–0.64, P_H_ = 0.470) (**Figure 3A**); CT versus CC: OR = 0.21 (95% CI: 0.07–0.62, P_H_ = 0.474); TT+CT versus CC: OR = 0.21 (95% CI: 0.07–0.62, P_H_ = 0.474)] (**Table 2**). What’s more, all three genetic models showed heterogeneity among Asians and we applied a random-effects model in Asian subgroup. No association with significance between *PTPN22*-C1858T polymorphism and probability to *M. tuberculosis* infection in Asians [C versus T: OR = 0.21 (95% CI: 0.05–0.82, P_H_ = 0.739) (**Figure 3A**); CT versus CC: OR = 0.21 (95% CI: 0.05–0.81, P_H_ = 0.733); TT+CT versus CC: OR = 0.21 (95% CI: 0.05–0.81, P_H_ = 0.733)] (**Table 2**).

### *PTPN22*-C1858T Polymorphism Correlated With Increased Susceptibility to *M. leprae* Infection

The relevance between *PTPN22*-C1858T polymorphism and increased possibility to *M. leprae* infection in Caucasian and Asian populations was investigated in two studies (17, 25). Owing to limited quantity of studies and heterogeneity (*P* = 0.108 and I-squared = 61.2%) (**Figure 3B**), among the study populations, we reviewed carefully with the results and adopted random-effects model in allele (C versus T), heterozygote (CT versus CC), and dominant (TT versus CT+CC) models for meta-analysis. A significantly increased risk of *M. leprae* infection was observed in the allele model [C versus T: OR = 2.82 (95% CI: 1.02–7.81, P_H_ = 0.108)] (**Figure 3B**) (**Table 2**). An elevated risk of leprosy was perceived in patients with *PTPN22*-C1858T polymorphism in heterozygote models [CT versus CC: OR = 2.91 (95% CI: 0.99–8.60, P_H_ = 0.094)] and dominant models [TT/CT versus CC: OR = 2.91 (95% CI: 0.99–8.60, P_H_ = 0.094)] (**Table 2**).

### Meta-Regression Was Applied to Analyzing Heterogeneity

Ethnicity and pathogen were included in meta-regression model for heterogeneity analysis. The coefficient of ethnicity is -0.060 (P = 0.955) and the coefficient of pathogen is 2.560 (P = 0.060) in all patients with Mycobacterial infection (**Table 3**), which indicated that pathogen (*M. tuberculosis/M. leprae*) was able to account for the heterogeneity of the associations between mycobacterial infections and *PTPN22*-C1858T polymorphism.

**Table 3:** Multivariate meta-regression analysis of PTPN22-C1858T in patients with mycobacterium infection.

Parameter	Coefficient	P value
Ethnicity	−0.060	0.955
Pathogen	2.560	0.060*

Ethnicity included Caucasian and Asian.

Pathogen included mycobacterium tuberculosis and Mycobacterium leprae.

*P < 0.1.

### Publication Bias

**Figure 4:**
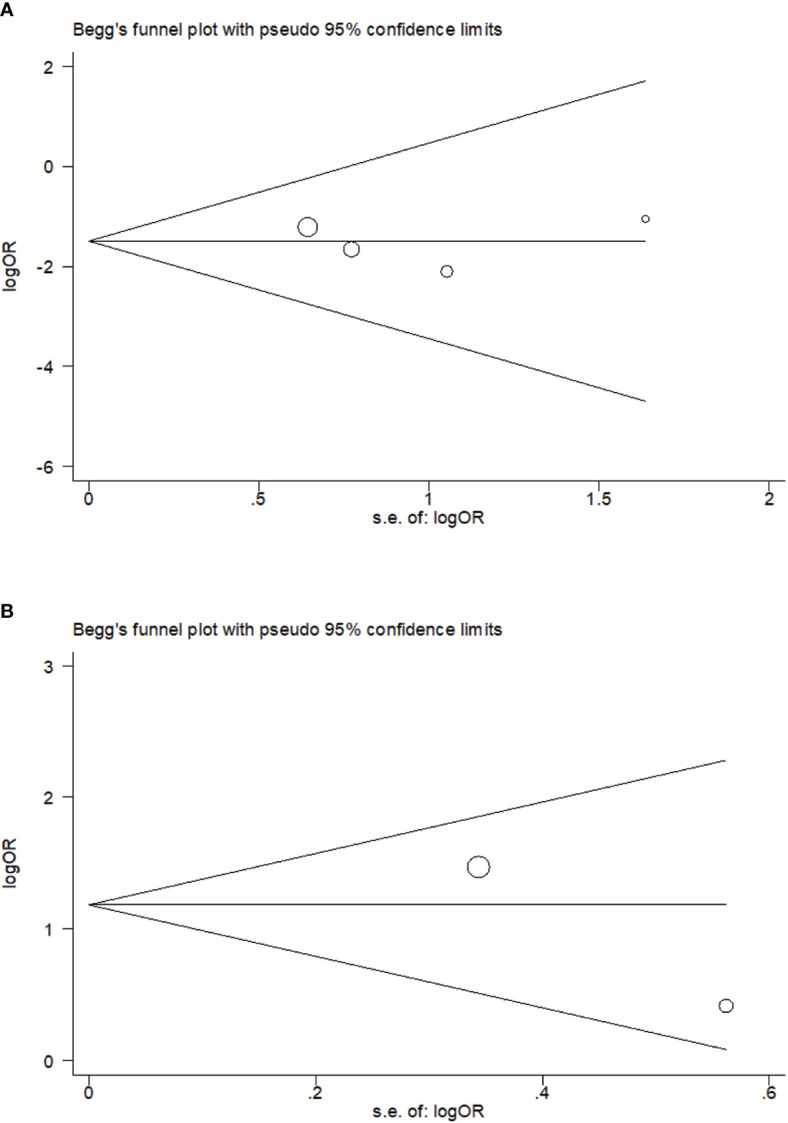
Begg’s funnel plot of rs2476601 (PTPN22-C1858T) polymorphism in individuals with *Mycobacterium* tuberculosis **(A)** and M. leprae **(B)** infection (C versus T).

No publication bias on relavance between PTPN22-C1858T polymorphism and possibility to tuberculosis (*P* = 0.734) or leprosy (*P* = 1.000) was reflected in Begg’s funnel plot. Meanwhile, we obtained a symmetrical funnel plots (**Figure 4**).

### Sensitivity Analysis

In order to determine impact from each study on pooled ORs for PTPN22-C1858T polymorphism, we performed sensitivity analysis by the means of deleting one of single study each time in every genetic model. The pooled ORs of both four studies related to M. tuberculosis infection (**Figure S1A**) and two studies related to M. leprae infection (**Figure S1B**) showed no significant association with PTPN22-C1858T polymorphism in above mentioned genetic models.

## Discussion

In this meta-analysis, we analyzed four eligible case-control studies including 595 subjects with tuberculosis and 697 controls and two studies including 306 subjects with leprosy and 562 controls. Our results indicate that *PTPN22*-C1858T polymorphism is uncorrelated with increased susceptibility to *M. tuberculosis* infection both among Caucasian and Asian populations in all genetic models but is related to increased susceptibility to *M. leprae* infection. However, further studies are required to validate the relevance between *PTPN22*-C1858T polymorphism and leprosy because of the limited quantity of studies and indicated heterogeneity among studies.

In addition to mycobacteria, a number of studies have focused on the relevance of *PTPN22*-C1858T polymorphism with raised susceptibility to infections by other bacteria (16, 28, 29). First, it was confirmed that individuals with *PTPN22*-C1858T polymorphism are predisposed to invasive infections with *Streptococcus pneumoniae* (16). Second, allogeneic hematopoietic stem cell transplant recipients have been found to have a significantly lower risk of post-transplantation bacterial infections after accepting corresponding allografts from *PTPN22*-C1858T polymorphic donors (28). Third, *PTPN22*-C1858T polymorphic patients with chronic mucocutaneous candidiasis showed a higher incidence of bacterial pulmonary infections than normal controls (29). Moreover, other *PTPN22* SNPs such as rs33996649 (*PTPN22*-G788A) might be related to raised susceptibility to tuberculosis. One study showed 788A allele was with higher frequency of in Moroccan patients with tuberculosis than in healthy donors [3.65% versus 0.65%, respectively; OR = 5.85 (95% CI:1.17–39.55, *P* = 0.01)] (19).

Detrimental effect of *PTPN22*-C1858T polymorphism-induced immune dysregulation on delayed-type hypersensitivity of tuberculosis and leprosy has been extensively studied. Herein, we discussed the relevance of *PTPN22*-C1858T polymorphism and increased risk of tuberculosis and leprosy in humans (17–19, 25–27). The *PTPN22* gene encodes for LYP, which render an impact on negative regulation to immunity (30). Moreover, *PTPN22*-C1858T carriers have variable immune patterns (31). Under physiological conditions, LYP selectively inhibits positive selection during a self-tolerant and immunological T-cell repertoire’s formation (32, 33), dephosphorylates the suppressive tyrosine located at Src family kinases’ C-terminus, and downregulate T-cell receptor (TCR) signaling by combining with growth factor receptor-bound protein 2 adapter (34–37). Patients with *PTPN22*-C1858T polymorphism show decreased calcium mobilization that is TCR-induced (38), reduced TCR-CD3ζ, Zap-70 and Lck’s phosphorylation (39), and decreased interleukin-2 production (39). However, some of these studies have yielded inconsistent conclusions. The absolute expression of *PTPN22* and proportion of T-cell subsets should be considered during assessment of the overall effect of *PTPN22*-C1858T polymorphism in T-cell-mediated immunity (40). Furthermore, increased susceptibility to mycobacterial infections is closely correlated with the immune status and genetic milieu of hosts. Most healthy individuals with a normal functioning immune system are resistant to *M. tuberculosis* or *M. leprae* and do not develop clinical tuberculosis or leprosy (41, 42). Host genetics accounts for a large proportion of inherited mycobacterial diseases; epidemiological investigations and functional genetic studies have identified several genes involved in mycobacterial infection. These studies provide information about the inherited predisposition to these infections (43–45). Hence, we conducted this meta-analysis to determine and articulate the relevance between *PTPN22*-C1858T polymorphism and risk of tuberculosis and leprosy.

Increased intracellular expression of LYP in patients with mycobacterial infection (46, 47), the involvement of *PTPN22* in downregulation of T-cell function (30), and the participation of *PTPN22* in invasive bacterial infection (16) have been considered as evidence to conclude that *PTPN22*-C1858T polymorphism increases the risk of mycobacterial disease onset (17). Moreover, *PTPN22* status has been shown to cause lymphoproliferative diseases in animal models. Splenomegaly and lymphadenectasis accompanied by initiative germinal center formation and higher-level antibodies are subtle immune changes observed in *PTPN22*-knockout mice (32). In addition, *PTPN22* promotes K63-linked polyubiquitination of the Toll-like receptor signal central promoter TRAF3 to upregulate type I interferons (IFNs) and promote type I IFN-dependent biological effects, causing immune cells to trigger a host defense response. In contrast, *PTPN22*-C1858T carriers show decreased TRAF3 K63-linked polyubiquitination and type I IFN production (48).

Roles of *PTPN22*-C1858T in infection and prevention mainly are beneficial to the patients with autoimmune diseases. *PTPN22*-C1858T is more often discussed in autoimmune disease. With the indispensable treatment with glucocorticoids, patient with autoimmune disease are suffering the from the risk of various infection. Additionally, we tend to claim that population with *PTPN22*-C1858T is more likely to gain autoimmune disease and the diseases itself carry a high risk of various infection. To this analysis, we are expecting our conslusions are able to provide useful informations for human to evade the potential infection and suggestion for the usage of immunosuppressors, especially for patients with autoimmune disease. To be more concrete, patients with *PTPN22*-C1858T are recommand a lower dose of immunosuppressors after diagnosis of autoimmne disease and they also are advised to notice all possiablity of infection.

We would like to emphasize a couple of strengths in our meta-analysis. First, we applied subgroup analysis to demonstrate the differences in the correlation between *PTPN22*-C1858T polymorphism and increased risk of mycobacterial infection. What’s more, the conclusion was verified in meta-regression model. Additionally, two independent investigators searched literatures without limitations so that we well controlled the selection bias. Besides, the result from Begg’s funnel plot revealed no evident publication bias, which render reliability to our conclusions. Finally, most of each studies in our meta-analysis attained a score representing high quality with regards to NOS quality assessment.

Nevertheless, we also acknowledge several limitations in this meta-analysis. In the first place, other factors contributing to mycobacterial infection were not considered due to the availability of limited information. Second, the results of *M. leprae* were obtained from two studies. Third, with a low allele frequency less than 1%, *PTPN22*-C1858T is a rare SNP in Asian population. Therefore, *PTPN22*-C1858T polymorphism would be rarely detected in most populations except Caucasians of Northern European descent (49). Fourth, the absence of cases and controls with TT in the six included studies may be attributed to their low survival rate for gene mutations. Fifth, tuberculosis and leprosy are considered curable diseases worldwide, especially in developed regions. Moreover, the annual incidence rate of these disease is declining. Furthermore, the diagnosis of leprosy is often not considered outside leprosy-endemic areas (50). Finally, the occurrence of a latency period following infection (42) and lengthy incubation periods of *M. leprae* (from a month to over 40 years) (50) might have excluded individuals with recessive infection or no infected (in the incubation period) to be registered as positive cases or were registered as controls.

In conclusion, we manifested that this *PTPN22*-C1858T polymorphism was uncorrelated with raised susceptibility to *M. tuberculosis* in Caucasians and Asians. In contrast, *PTPN22*-C1858T polymorphism related to increased susceptibility to *M. leprae* infection. However, the results of M. leprae are supposed to interpreted with. Further well-designed studies with sufficient populations are required to verify our conclusions.

## Data Availability Statement

The original contributions presented in the study are included in the article/**Supplementary Material**. Further inquiries can be directed to the corresponding authors.

## Author Contributions

YC and SL conceived the topic and analyzed data. SL wrote this manuscript and YC modified it. JY provided statistical suggestions. SL and YZ evaluated the quality of the six included studies. SL, YZ, XW, and TC searched references.Y, ZJZ, and ZZ provided the overall direction of series studies and funding support. All authors contributed to the article and approved the submitted version.

## Funding

This work was supported by the 100 Top Talents Program of Sun Yat-sen University, National Natural Science Foundation of China (NSFC, Grant No. 31871400), the Shenzhen Science and Technology Innovation Committee of Guangdong Province of China (Grant No. JSGG20180703155802047), and the Shenzhen Science and Technology Innovation Committee of Guangdong Province of China (Grant No. JCYJ20180307150634856).

## Conflict of Interest

The authors declare that the research was conducted in the absence of any commercial or financial relationships that could be construed as a potential conflict of interest.

## Abbreviations

CI, confidence interval; CNKI, Chinese National Knowledge Infrastructure; HWE, Hardy-Weinberg equilibrium; IFN, interferon; NOS, Newcastle-Ottawa Scale; OR, odds ratio; PTPN22, protein tyrosine phosphatase non-receptor type 22; SNP, single-nucleotide polymorphism; TCR, T-cell receptor.
