# Supplementary material for: Association of PTPN22-C1858T Polymorphism With Susceptibility to Mycobacterium tuberculosis and Mycobacterium leprae Infection: A Meta-Analysis
Source: Front Immunol. 2021 Feb 25;12:592841. doi: 10.3389/fimmu.2021.592841 (PMC7950544; doi:10.3389/fimmu.2021.592841)
Supplement: Supplementary file 1 [file DataSheet_1.pdf]

Supplementary Material

Supplement 1

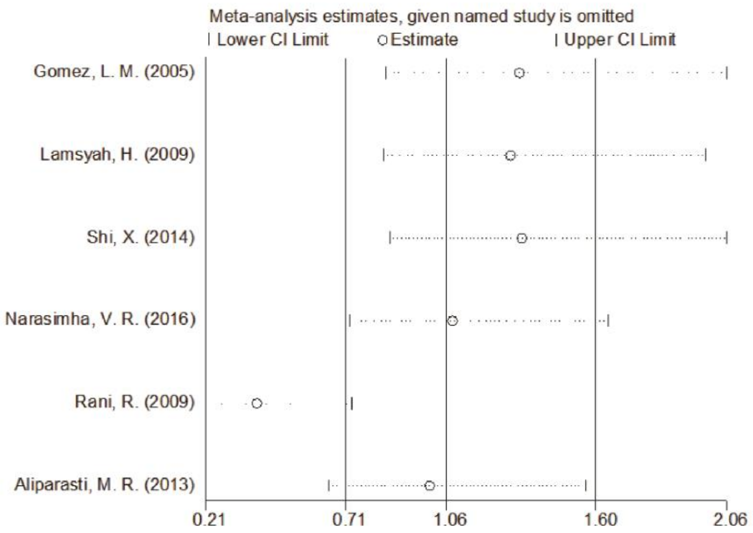

a

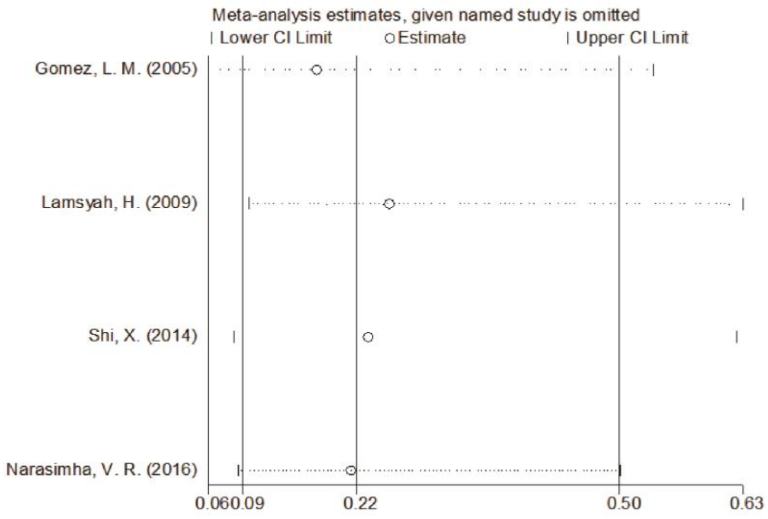

b

Supplementary Figure 1. Sensitivity analysis for the effect of each study on pooled ORs for the correlation between susceptibility to M. tuberculosis (a) and M. leprae (b) infection and rs2476601 PTPN22-C1858T polymorphism (C vs. T).
