# Supplementary material for: Association of PTPN22-C1858T Polymorphism With Susceptibility to Mycobacterium tuberculosis and Mycobacterium leprae Infection: A Meta-Analysis
Source: Front Immunol. 2021 Feb 25;12:592841. doi: 10.3389/fimmu.2021.592841 (PMC7950544; doi:10.3389/fimmu.2021.592841)
Supplement: Supplementary file 3 [file DataSheet_3.pdf]

Association of *PTPN22*-C1858T polymorphism with susceptibility to *Mycobacterium tuberculosis* and *Mycobacterium leprae* infection: a meta-analysis

Table S1. Newcastle-Ottawa quality assessment scale for six case-control studies included in the meta-analysis.

First author, year	Selection				Comparability		Exposure			Quality
	Case Definition	Representativeness of cases	Selection of Controls	Definition of Controls	The most important factor	Additional factor	Ascertainment of exposure	Same method of ascertainment for cases and controls	Non-Response rate	
Gomez, L. M., 2005	a	a	c	a	race	yes	b	a	a	8
Lamsyah, H., 2009	a	a	c	a	sex	yes	b	a	a	8
Shi, X., 2014	a	a	b	a	no	yes(age)	b	a	a	7
Narasimha, V. R., 2016	a	a	c	a	no	no	b	a	a	6
Rani, R., 2009	a	a	c	a	race	no	b	a	a	7
Aliparasti, M. R., 2013	a	a	a	a	no	no	b	a	a	7
